# Supplementary material for: Comparative multiomics analysis of cell physiological state after culture in a basket bioreactor
Source: Sci Rep. 2022 Nov 23;12:20161. doi: 10.1038/s41598-022-24687-4 (PMC9686226; doi:10.1038/s41598-022-24687-4)
Supplement: Supplementary file 1 — Supplementary Information 1. [file 41598_2022_24687_MOESM1_ESM.zip › raw data/Metabolomics raw data/5.MetKeggEnrichment/EG.vs.CG/EG.vs.CG_neg.KEGG_Enrich.scatterplot.pdf]

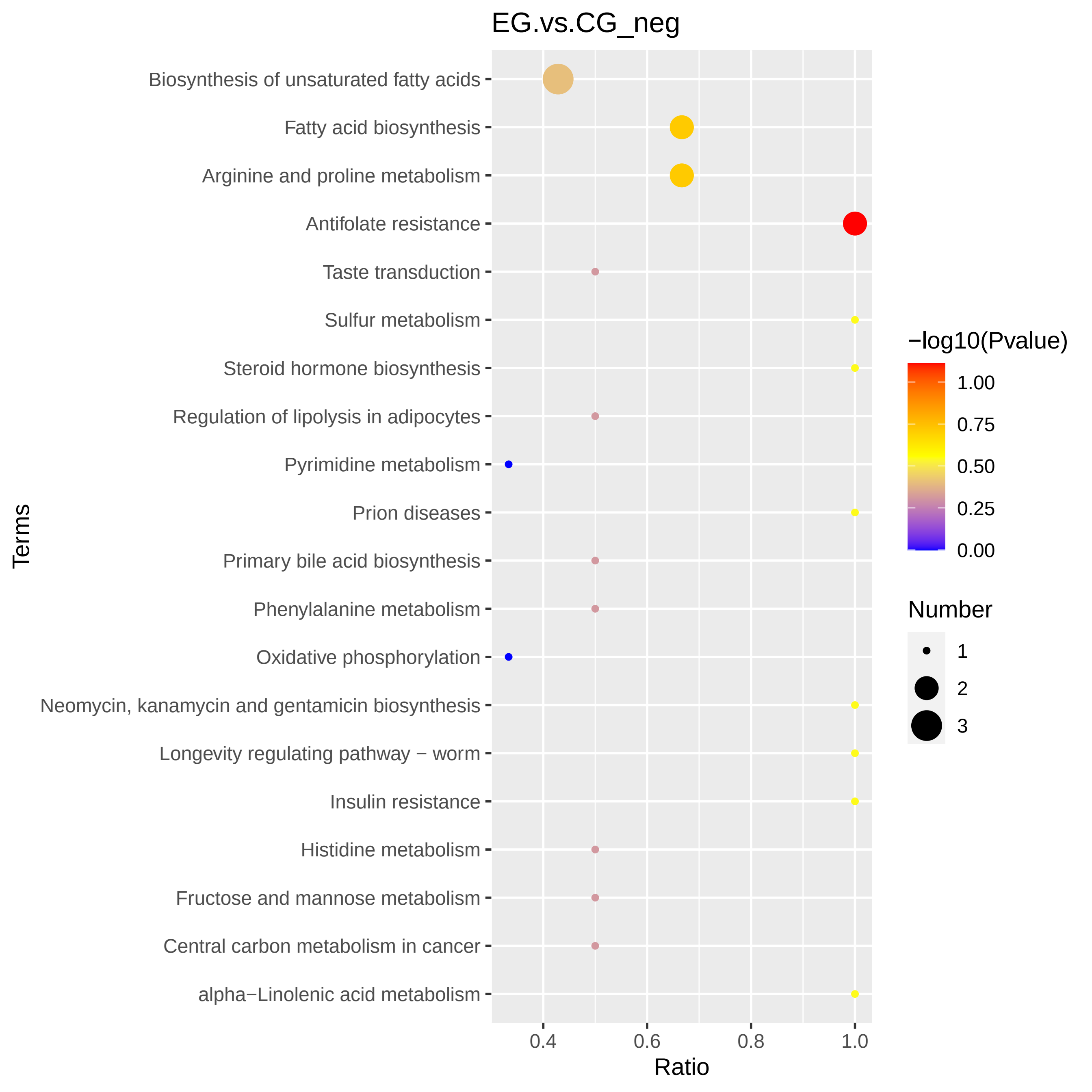
EG.vs.CG_neg
Biosynthesis of unsaturated fatty acids
Fatty acid biosynthesis
Arginine and proline metabolism
Antifolate resistance
Taste transduction
Sulfur metabolism
Steroid hormone biosynthesis
Regulation of lipolysis in adipocytes
Pyrimidine metabolism
Prion diseases
Primary bile acid biosynthesis
Phenylalanine metabolism
Oxidative phosphorylation
Neomycin, kanamycin and gentamicin biosynthesis
Longevity regulating pathway − worm
Insulin resistance
Histidine metabolism
Fructose and mannose metabolism
Central carbon metabolism in cancer
alpha−Linolenic acid metabolism
Terms
0.4
0.6
0.8
1.0
Ratio
−log10(Pvalue)
1.00
0.75
0.50
0.25
0.00
Number
1
2
3
